# Priorities for Breast Cancer Research: Taking Stock of Chemicals, Biomarkers, and Exposure Assessment Tools

**DOI:** 10.1289/ehp.122-A253

**Published:** 2014-09-01

**Authors:** Nancy Averett

**Affiliations:** Nancy Averett writes about science and the environment from Cincinnati, OH. Her work has been published in *Pacific Standard*, *Audubon*, *Discover*, *E/The Environmental Magazine*, and a variety of other publications.

Inherited genetic factors by themselves contribute relatively little to breast cancer risk; for many patients, researchers believe, environmental factors are likely to play a role in the development of the disease.^1^ Yet, few epidemiological studies have focused on chemical compounds vis-à-vis breast cancer risk due, in part, to a lack of information on which chemicals to pursue and how best to measure exposures.^2^^,^^3^ In a review in this issue of *EHP*, researchers at Silent Spring Institute examine which chemicals are of greatest concern as potential risk factors for breast cancer as well as methods for quantifying exposure to these chemicals.^4^

After conducting a systematic literature review, lead author Ruthann Rudel and colleagues selected 102 chemicals linked with mammary tumors in rodent studies. The authors limited the list to chemicals that people are likely to encounter, either through occupational contact, through air, food, or consumer products, or through pharmaceuticals that are used by large numbers of women or targeted to pregnant women. The authors uncovered published methods for measuring exposure to 73 of these 102 chemicals.^4^

**Figure:**
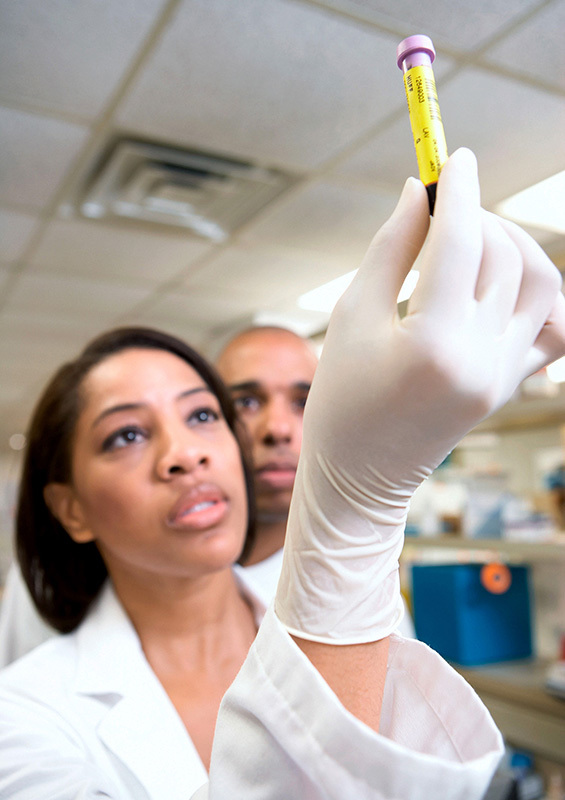
Archived samples of blood, urine, and other tissues collected through cohort studies could aid our understanding of human breast carcinogens. © Blend Images/Alamy

For 19 of the chemicals, there was also evidence of carcinogenicity in humans. Major reviews evaluated by the authors indicated animal studies are, in general, good predictors of breast carcinogenicity in humans.^4^

Based on these evaluations, the authors identified 17 chemicals or groups of chemicals they deemed to be the highest priorities for breast cancer research. Among them are benzene (found in gasoline, tobacco smoke, and some consumer products), perfluorooctanoic acid (found in nonstick and stain-resistant coatings), and pharmaceutical hormones. Priority was based on the strength of the evidence for carcinogenicity, the likelihood of human exposure, and the existence of measurement methods.^4^

“There’s quite a lot of biological evidence that chemicals are plausibly linked to breast cancer, but very little research has been focused on this,” says coauthor Julia Brody. “We wanted to really open up the discussion about breast cancer prevention and provide a road map for considering chemicals that are mammary gland carcinogens in rodents.”

Rudel and colleagues also compiled a list of potentially relevant cohort studies for which archival samples might be available for exposure biomarker testing. Biomarkers are chemicals or metabolites that can be measured in biological samples, most commonly in blood and urine, but also in milk, exhaled breath, hair, saliva, fingernails, and fat. In all, they identified 60 candidate cohort studies encompassing more than 3.5 million women and girls.^4^

“It’s an incredibly comprehensive review. It pulls together a lot of information that those who don’t usually study chemicals would find very useful as a starting place,” says Dale Sandler, director of the NIEHS Sister Study,^5^ which is a cohort of more than 50,000 women whose sisters have been diagnosed with breast cancer. Sandler cautions, however, that although the identification of measurement methods is a great resource, it does not mean researchers can immediately start studying exposures in their cohorts.

She explains that investigators will have to gather preliminary evidence about exposure ranges and opportunities in the cohort to prioritize which chemicals to study and before deciding whether to use up precious samples and invest in costly assays. This might involve pilot studies of banked samples and questionnaires in which cohort participants self-report their exposures to chemicals at certain points in their lifetimes.

Dora Il’yasova, an epidemiologist at Georgia State University with expertise in biomarker-based exposure assessments, agrees that measuring biomarker exposures can be tricky and thus requires careful consideration before investigators jump in. As an example, she cites the transient nature of most biomarkers, which complicates their detection in bodily fluids. “As always,” she says, “the devil is in the details.”
